# Supplementary material for: A meta-analysis suggests that TMS targeting the hippocampal network selectively improves episodic memory
Source: eLife. 2026 May 14;14:RP108934. doi: 10.7554/eLife.108934 (PMC13175575; doi:10.7554/eLife.108934)
Supplement: Supplementary file 1. [file elife-108934-supp1.docx]

| # | Factor | Levels | Description | Rationale |
| --- | --- | --- | --- | --- |
| 1 | Task | Recognition | Episodic memory tests using recognition formats; i.e., memoranda are repeated from study to test and subjects make old/new discrimination judgments | Recollection is thought by many to be more heavily dependent on hippocampus and its network than familiarity, which differentially contribute to tasks using recollection and recognition formats. While Recollection tasks could have been sensitive to many types of memory processing, including familiarity, at least one aspect of performance depended on recollection. Performance of tasks with other formats could be supported by an unknown mix of recollection, familiarity, or other processes. |
|  |  | Recollection | Episodic memory tests using recollection formats; i.e., free recall, cued recall, or self-reported recollection judgments for tasks using paired associates, item-context associates, or multi-part complex stimuli as memoranda |  |
|  |  | Other | Episodic memory tests using other formats; i.e., temporal order judgments, recall of details from videos, scored visual reproduction, and tests that assess mnemonic pattern separation |  |
| 2 | Targeting | Individual | The location where TMS was applied was determined by subject-specific analysis of functional or structural MRI connectivity of hippocampus; i.e., a location of parietal/occipital neocortex was targeted based on a maximal connectivity value with hippocampus defined in each subject. | Individualized targeting has been used in many studies based on the assumption that network structure varies across people and that indirect influences of stimulation on the hippocampus are transsynaptic and predicted by connectivity. Group targeting assumes that there is sufficient homogeneity across subjects to reliably affect networks, or was based on hypotheses about specific parietal areas that happen to be among hippocampal network areas typically targeted using individualized targeting approaches. |
|  |  | Group/atlas | TMS was applied to approximately the same target in each subject, based on functional or structural MRI connectivity in a group/atlas or based on a specific hypothesis about the function of a specific location. |  |
| 3 | HITS Timing | Pre-task | TMS was delivered sometime before the period of memory encoding, ranging from application during the several seconds before individual memoranda were studied, in the minutes to hours before an encoding block was administered, to for many sessions over several days or weeks before the encoding phase of the memory task was completed. | This is a post hoc factor. Most studies used the "before" design whereas some used the "after" design. Hippocampal involvement in memory could differ for encoding versus retrieval, although an important caveat is that the timecourse of stimulation effects on brain activity and memory is not known, and so claims of temporal specificity cannot be assessed. |
|  |  | Post-encoding | All of these experiments applied a single session of stimulation after a block of encoding and before the corresponding memory test was given minutes to tens of minutes later. |  |
| 4 | Population | Younger adult | Young adults with no reported neurologic or psychiatric disorders were participants (<45 years old). | Populations with age-related memory decline (older adult) or with clinical memory impairments (MCI/AD and potentially Other) could present more opportunity for stimulation to improve memory. Alternatively, network disruptions frequently observed in memory disorders could reduce the ability for TMS to indirectly influence hippocampus due to reduced possibility of transsynaptic influence. Thus, the a priori hypotheses are that effects will differ among populations. |
|  |  | Older adult | Older adults with no reported neurologic or psychiatric disorders were participants (>65 years old). |  |
|  |  | MCI / AD | Participants included individuals with diagnoses of Mild Cognitive Impairment (MCI) or mild to moderate Alzheimer's dementia (AD) following clinical standards. |  |
|  |  | Other | Other groups included individuals with first-episode psychosis, individuals at high risk of schizophrenia, and individuals with symptoms of anxiety disorders of post-traumatic stress disorder. |  |
| 5 | Target | Left parietal | Lateral parietal cortex (LPC) was stimulated in left hemisphere in most studies, at locations defined via either individualized or group targeting methods, as shown in Figure 1. | This is a post hoc factor. The majority of studies targeted areas of lateral, inferior, posterior parietal cortex near the angular gyrus, following the general method of Wang et al. 2014. Others targeted precuneus or other parietal or anterior occipital areas based on connectivity with hippocampus. All of these areas are considered within the hippocampal network and so no strong a priori hypotheses regarding the effects of HITS were warranted. |
|  |  | Other | Other stimulation locations included precuneus or other parietal-occipital areas defined based on individual or group targeting methods, as shown in Figure 1. These were combined because separating them would have led to very few effects in each separate level. |  |
| 6 | Task delay | Short | The effects of stimulation were measured on the same day on which stimulation was delivered. Some studies applied short stimulation trains in the seconds before memoranda were presented, others delivered a several-minute stimulation train and then tested effects within one hour, and others used multi-day or multi-week stimulation and tested the effects on the same day following the last stimulation session. | This is a post hoc factor. Generally, the effects of stimulation are thought to decay, albeit at unknown rates. However, studies tended to measure outcomes at test delays aligned with the duration of stimulation (i.e., shorter test delays for protocols involving less stimulation and longer test delays for protocols involving more stimulation over more days; see Figure S2). Thus, as test delay was aligned to measure outcomes appropriately for hypothetical protocol-specific hypothesized decay rates, there was no strong a priori hypothesis to suggest that test delays would be non-equivalent. |
|  |  | Medium | The effects of stimulation were measured on the following day after stimulation delivery. These studies mostly delivered several consecutive daily sessions of stimulation and measured the effects ~24 hours after the final stimulation session. |  |
|  |  | Long | The effects of stimulation were measured more than one day after the final stimulation session in multi-day or multi-week stimulation protocols, up to ~4 weeks after the end of stimulation. |  |
| 7 | Sessions | 1 | Protocols involved only one session of stimulation before outcomes were assessed. This encompasses studies that used short trains of stimulation (seconds) immediately before memoranda were presented, as well as several-minute trains of stimulation before memory was assessed. | This is a post hoc factor. Generally, more sessions of stimulation are thought to produce greater and/or longer-lasting effects. However, as session counts and task delays were correlated (see “Task delay” factor), there were no strong a priori hypotheses about differences among session counts. |
|  |  | 2 – 4 | Protocols involved two to four consecutive sessions of stimulation spread out across multiple days. |  |
|  |  | 5+ | Protocols involved five or more sessions of stimulation spread out across multiple days and/or weeks |  |
| 8 | Intensity | 100+% MT | Stimulation was applied at an intensity of greater than or equal to the resting motor threshold (MT), typically no higher than 120% of the MT. | This is a post hoc factor. Generally, higher intensities would be thought to have greater effects. However, this factor overlapped considerably with TMS protocol (factor 11, which was not retained in the lasso regressions). That is, studies using repetitive TMS trains at high frequencies tended to use intensities at or above motor threshold, whereas studies using patterned theta-burst stimulation tended to use intensities lower than 100% of motor threshold (Figure S2). Thus, there were no strong a priori hypotheses concerning this factor specifically. |
|  |  | <100% MT | Stimulation was applied at an intensity of less than 100% of the resting MT, typically at 90%, 80%, or 70% of the MT. Note that one study (study 8) used 80% of active MT, rather than resting MT. |  |
| 9 | Control | Baseline | The comparison for the effects of stimulation was the baseline, pre-stimulation performance value. | This was a post hoc factor. Different control conditions are used to achieve different scientific objectives. As all control conditions would be expected to yield little if any effect on memory performance, any significant variation in effects of HITS on memory by control condition would raise doubts about its robustness to small differences in experiment design choices. |
|  |  | Other location | The comparison for the effects of stimulation of the hippocampal network was stimulation of some other part of the brain not within the hippocampal network using the same stimulation parameters. Typically, primary or secondary motor or somatosensory areas were used as control locations. |  |
|  |  | Low intensity | The comparison for the effects of stimulation was stimulation of the same location using the same overall stimulation parameters but at a very low intensity, typically <30% of resting motor threshold. |  |
|  |  | Other | The comparison for the effects of stimulation was stimulation of the same location using a different rhythmic pattern that was hypothesized to have less of an effect on memory. |  |
| 10 | Design | Post - Post | The effects of stimulation on performance were statistically compared for after receiving HITS versus after receiving the control condition (e.g., t test of the performance scores after HITS versus after control). | This was a post hoc factor. Different statistical designs are used to achieve different scientific objectives and to accommodate different practical constrains on experiment design. As the choice of statistical design would be expected to yield little if any effect on memory performance, any significant variation in effects of HITS on memory by analytic design would raise doubts about its robustness to small differences in experiment analysis choices. |
|  |  | Pre - Post | The statistical comparison of effects considered performance after versus before receiving HITS (e.g., t test of performance scores after HITS versus baseline before HITS). |  |
|  |  | Delta | The statistical comparison of effects on performance was done by computing the pre- versus post-HITS change (delta) in scores and comparing to the pre- versus post-Control delta (e.g., t test of percentage change baseline to post-HITS versus percentage change baseline to post-Control). |  |
|  |  | Interaction | The statistical test of effects on performance treated time (pre- versus post-) and stimulation type (HITS versus control) as factors, as in a RM-ANOVA. |  |
|  |  | Other | More than two stimulation conditions, including the control condition, were compared as factors, as in a RM-ANOVA |  |
| 11 | TMS protocol | rTMS | rTMS (1, 10, 15, or 20 Hz) applied in either short trains of several seconds, minutes, or as a longer, approximately 20-min trains per session. Note that only two studies used 1 Hz rTMS whereas all others used 10Hz or higher. | Some evidence suggests that theta burst stimulation has greater effects on hippocampal network activity and episodic memory performance. However, this factor was strongly correlated with Intensity and Sessions factors (Figure S2). NOTE: this factor was not retained in the lasso regressions as it was strongly correlated with other factors, thus warranting no strong a priori hypotheses of its independent contributions. |
|  |  | TBS | Theta-burst stimulation, including short trains of several seconds of theta burst, or as continuous theta burst (40 s) or intermittent theta burst (5 minutes) sessions. |  |

**Supplementary File 1. Table providing description of study factors.**
